# Remote microphone systems in children and adolescents with autism spectrum disorders: a scoping review

**DOI:** 10.1590/2317-1782/e20230310en

**Published:** 2025-02-24

**Authors:** Bianca Stephany Barbosa Vital, Karen Melissa Gonzaga dos Santos, Aryelly Dayane da Silva Nunes Araújo, Joseli Soares Brazorotto, Regina Tangerino de Souza Jacob, Karinna Veríssimo Meira Taveira, Sheila Andreoli Balen

**Affiliations:** 1 Programa Associado de Pós-graduação em Fonoaudiologia, Universidade Federal do Rio Grande do Norte – UFRN - Natal (RN), Brasil.; 2 Universidade Federal da Paraíba – UFPB - João Pessoa (PB), Brasil,; 3 Universidade Estadual de Ciências da Saúde de Alagoas – UNCISAL - Maceió (AL), Brasil.; 4 Laboratório de Inovação Tecnológica em Saúde – LAIS, Universidade Federal do Rio Grande do Norte – UFRN - Natal (RN), Brasil.; 5 Departamento de Fonoaudiologia, Universidade Federal do Rio Grande do Norte – UFRN - Natal (RN), Brasil.; 6 Departamento de Fonoaudiologia, Faculdade de Odontologia de Bauru – FOB, Universidade de São Paulo – USP - Bauru (SP), Brasil.; 7 Departamento de Morfologia, Universidade Federal do Rio Grande do Norte – UFRN - Natal (RN), Brasil.

**Keywords:** Autism Spectrum Disorder, Remote Microphone, Auditory Perception, Children, Teens

## Abstract

**Purpose:**

To map the literature on the use of the Remote Microphone System (RMS) in children and adolescents with Autism Spectrum Disorder (ASD).

**Methods:**

Scoping Review following the Joanna Briggs Institute recommendations and PRISMA-ScR checklist. Search was carried out in the databases: PubMed, Embase, Scopus, Web of Science, Lilacs, and gray literature, including Google Scholar and ProQuest, as well as reference lists of included studies and expert consultations. Intervention studies with children and adolescents with ASD using RMS were included, without gender, language, age, publication time, ethnicity, or geographical location restrictions.

**Results:**

709 studies were identified in phase 1. After reviewing 14 full texts with eligibility, eight studies were eligible. Studies were heterogeneous in the RMS model (personal or free field), applied tests, intervention period, and location. Improvement in speech perception, social interaction, behavior, attention, auditory memory, noise tolerance, stress reduction, and modification in neural activity through electrophysiological evaluation were observed.

**Conclusion:**

Using RMS demonstrated benefits in speech perception, social interaction, and behavior in adolescents and children with ASD. Further studies are needed to define protocols and indication parameters in this population.

## INTRODUCTION

In classrooms, environmental noises take many different forms, such as conversations between students, the movement of chairs and desks, the crinkling of notebooks, the sounds of the fan and air conditioning, the corridor and the street, and through the window, among many other noises that can make it difficult for the teacher to speak^(1)^.

Children with Autism Spectrum Disorder (ASD) can be found in the school context. ASD is a neurodevelopmental disorder with a growing prevalence and which occurs in a heterogeneous way, being characterized by difficulties in social communication, repetitive behavior, focus of interest, and sensory alterations^(2)^.

These children may have persistent difficulties in communication and social interaction, as well as restricted and repetitive behavior patterns (whether behavioral or interests/activities). Interventions to help these children and adolescents develop their abilities and potential, whether in the school, family, or social environment, are essential^(2)^.

Research is being carried out on schoolchildren with ASD and the use of the remote microphone system (RMS) because they have listening difficulties. Studies^(3,4)^ have shown potential benefits when remote microphone systems are used in children and young adults^(3,4)^, with benefits in speech perception in the school environment after the intervention. The RMS is an assistive technology that has been widely studied and indicated in other populations, especially for individuals with hearing loss (HL)^(5-7)^ and central auditory processing disorder^(8-10)^.

The term RMS encompasses digital signal and frequency-modulated (FM) transmission technologies^(11)^, which can be used in person or in the field. The first is a device made up of a transmitter that picks up the acoustic signal from the main speaker (teacher) and transmits it by digital signal to the listener (student) who is using a receiver in the ear. This acoustic signal reaches the listener with reduced noise and a better acoustic signal and is for individual use^(11)^.

In the case of field RMS, the aim is collective use, where the voice of the main speaker is amplified and transmitted by a speaker in the field to a larger number of people, such as students in a classroom^(12)^. The Audiology Society of Australia^(13)^ has emphasized the importance of audiologists in the intervention of children with ASD by recommending RMS in various environments.

Given this panorama, the aim of this scoping review was to map the studies on the use of RMS in children and adolescents with ASD.

## METHODS

### Protocol and registration

This study is a scoping review following the methodological guidelines of the Joanna Briggs Institute (JBI) and the recommendations of the PRISMA-ScR checklist (Preferred Report items for systematic reviews and Meta-analyses extension of Scoping Reviews, 2018). This type of study uses a systematic approach to synthesize knowledge, identifying the main concepts, theories, sources, and gaps in knowledge^(14)^. The research protocol for this study was submitted for evaluation and registration on the Open Science Framework (OSF) platform, DOI: 10.17605/OSF.IO/Q4GBF.

### Eligibility criteria

The acronym ‘PCC’ was used to list the studies eligible for this review: P = Population (children and adolescents diagnosed with ASD); C = Concept (Evidence after intervention with Remote Microphone in children and adolescents with ASD); C = Context (use of the RMS).

### Inclusion criteria

Studies evaluating the use of RMS in children and adolescents with ASD without restrictions on gender, language, age, time of publication, ethnicity, or geographical location.

### Exclusion criteria

The following exclusion criteria were applied: participants with hearing loss, studies without pre- and/or post-intervention with RMS; studies with designs other than intervention; studies with children and adolescents without a diagnosis of ASD; studies with adults and/or the elderly; types of publications such as systematic reviews, meta-analyses, books, guidelines, websites, blogs.

### Literature search

The search strategy was first carried out on Pubmed and adapted for the other databases used in this review: Embase, Scopus, Web of Science, Lilacs, Google Scholar, and ProQuest, on 16 March 2023 (Appendix 1).

After searching each database, the results were exported to the Mendeley software, where duplicate articles were identified. The file was then saved and exported to the Rayyan website (https://www.rayyan.ai/), where duplicates were rechecked and two independent reviewers read the title and abstract.

### Study selection

The studies were selected in two phases. In the first phase, two reviewers independently analyzed the title and abstract based on the eligibility criteria. This stage was carried out on the Rayyan website. In the second phase, the full texts were read independently by the same reviewers. In both phases, a consensus meeting was held before finalizing the stage. When there was no consensus between R1 and R2 on the inclusion/exclusion of one or more studies, the third reviewer analyzed them to make a decision (Appendix 2).

**Figure 1:**
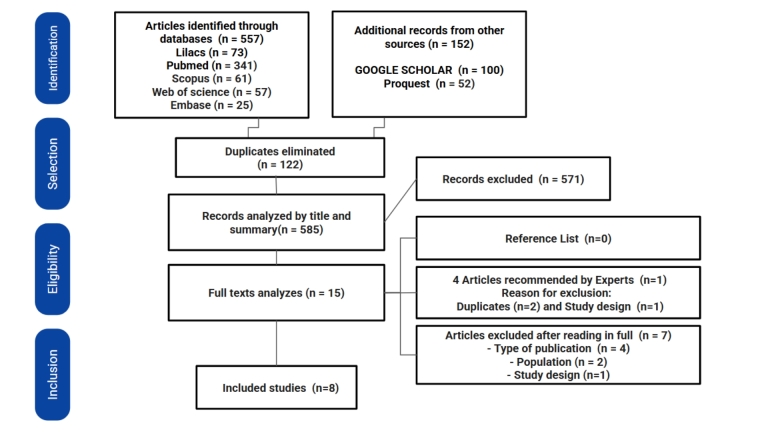
Flow diagram according to PRISMA-ScR guidelines (adapted)

Source: Prisma-ScR adaptation by the author

The reference lists of the studies included in phase 2 were analyzed, and three experts in the field were consulted to identify any studies that may have yet to be retrieved in the initial search. These experts were contacted because they are PhD researchers with published studies on the subject presented in this review from different countries such as Australia, New Zealand, and the United States. These experts were the authors of studies already identified in the database search. Of the four articles they indicated, only one was included, as two were duplicates and one did not meet the study design criteria, as can be seen in Figure 1.

### Data analysis and extraction

Data from the study was extracted, such as author, year of publication, country, objective, sample, age, ASD diagnostic tool, language level, pre-intervention audiological acuity, questionnaires/protocols (pre- and post-intervention), assistive technology, intervention process, and main outcomes.

All the data related to the scoping review was extracted and mapped, and a qualitative synthesis was carried out.

## RESULTS

### Study selection

The studies were selected as shown in Figure 1. A total of 709 studies were retrieved from the electronic databases, 557 from the database, and 152 from the gray literature. After insertion into the Mendeley software, 585 studies remained after duplicates were removed, which were then analyzed for title and abstract in the Rayyan software. Following the eligibility criteria, 571 articles were excluded, followed by 14, and one was inserted by expert recommendation, following a total of 15 articles for full reading in phase 2. Of these, 7 studies were excluded and 8 were included in the qualitative synthesis and mapping of results (Figure 1).

### Characteristics of the studies and the sample

The studies included were published between 2013 and 2021, five of them in the United States of America^(15-19)^, two in Australia^(20,21)^ and one in New Zealand^(22)^. The research groups are fourfold with studies by Schafer et al.^(15-17)^, Rance et al.^(20,21)^, Keller et al.^(18)^ , Keller^(19)^ e Leung et al.^(22)^.

Of the eight studies included, six^(15-19,22)^ carried out an intervention with a sample size of between 8 and 14 subjects. The studies from the Australian group^(20,21)^ had the largest sample size - 20 and 26 participants (Table 1). And in six studies, comorbidities associated with ASD were reported^(15,16,18,19,21,22)^.

**Table 1:** Sample characterization, confirmation of ASD diagnosis, expressive language modality and audiological acuity

Author/year/place	Sample (n)	Age (years)	ASD diagnosis	Language Status	Audiological status
Keller et al.^(18)^ (2021), United States of America	8	3-4	Researchers and/or multidisciplinary team	Verbal	Normal hearing
Keller^(19)^ (2021), United States of America	14	4-16	Researchers and/or multidisciplinary team	Verbal	Normal hearing
Rance et al.^(20)^ (2014), Australia	20	8-15	Researchers and/or multidisciplinary team	Verbal	Normal hearing
Rance et al.^(21)^ (2017), Australia	26	6-16	Multidisciplinary team	Verbal	Normal hearing
Schafer et al.^(15)^ (2013), United States of America	7	9-11	Parents and confirmed by the school administration	Verbal	Normal hearing
Schafer et al.^(16)^ (2014), United States of America	4	9-10	Informed by parents and confirmed by specialized professionals	Verbal	Normal hearing
Schafer et al.^(17)^ (2016), United States of America	12	6-17	Carried out by professionals and reported by parents	Verbal	Normal hearing
Leung et al.^(22)^ (2021), New Zealand	12	7-13	Researchers or multidisciplinary team	Verbal	Normal hearing

Source: Own authorship

As for the age range of the participants with ASD in the studies, only one^(18)^ involved preschoolers between 3 and 4 years old. Of the other studies, three were carried out with older children, aged between 7 and 13^(15,16,22)^, and in four studies the intervention was carried out with children and adolescents aged between 4 and 17^(17,19-21)^. Only one study^(21)^ had two groups divided by age, one with children aged 6 to 12 and one with children aged 13 to 16.

The inclusion criterion for the intervention was a confirmed diagnosis of ASD. In four studies^(18-22)^ the diagnosis was confirmed by the researchers or multidisciplinary team through instruments such as ADOS-2, Autism Diagnostic Interview, Childhood Autism Rating Scale, ASEBA, CARS-2, in which they had access to the assessment reports.

In three studies, confirmation of the diagnosis was provided by parents and/or the school (who made the reports available)^(15-17)^, and in one study^(15)^ the reports were not made available to the researchers.

The linguistic development of the subjects was not characterized by the authors, and there was heterogeneity in the reported linguistic characteristics of these children and adolescents, although it was clear that all the subjects were verbal. Two studies^(18,19)^ used the term “minimally verbal autism” (MVA) to describe the language of their participants who had single words and echolalia. In the first study, prior assessment was carried out using the Mullen Scales of Early Learning (MSEL) protocol to identify language delays/difficulties, and the second study defined classification using module 1 of the Autism Diagnostic Observation Schedule (ADOS-2) test.

In another study^(17)^, participants were classified with verbal skills after being categorized by the study examiners into five categories, namely^(1)^ echolalia and few spontaneous words^(2)^, one to three spontaneous words^(3)^, produces sentences with four or more words^(4)^, produces two or three sentences and^(5)^ are conversational. In the studies by Rance et al.^(20,21)^ all the participants in the intervention could speak, understand, and follow verbal instructions. In the studies by Schafer et al.^(15,16)^ it was possible to infer that the children were verbal from the application of the BKB-SIN test to assess speech recognition ability in noise. In the study by Leung et al.^(22)^, all the subjects had high-functioning autism, which suggests that the children were verbal.

Hearing acuity was previously assessed in the participants (Table 1). Only one study^(20)^ did not mention an evaluation but implied that the participants had hearing acuity within normal standards. One study considered the report of caregivers or medical records to be normal hearing^(19)^; another reported a retrospective analysis of medical records on the hearing acuity of at least one of the ears, highlighting that free-field measurements were only carried out on participants who did not agree to wear headphones^(18)^.

The studies by Shafer et al.^(15-17)^ and Rance et al.^(21)^ presented the following as inclusion criteria in their studies: hearing screening within the previous six months (in the frequencies of 1, 2, and 4 kHz at up to 25 dB); hearing assessment within normal standards (250 to 6000 Hz with thresholds below 20dB or presence of Transient Evoked Otoacoustic Emissions - TEOAE); hearing assessment at 250 to 8000 kHz or the presence of Distortion Product Otoacoustic Emissions (DPOAE); and threshold testing at 250 to 4000 kHz respectively, to confirm that the children and adolescents in the studies did not have hearing problems. In the study by Leung et al.^(22)^, the participants' hearing was assessed using DPOAE hearing screening.

### Pre- and post-intervention questionnaires and protocols

All the authors used pre- and post-intervention instruments to evaluate the RMS intervention process. A variety of functions/skills were assessed as a result of the different instruments used. Auditory recognition protocols^(15-21)^, questionnaires to assess: auditory functions/skills^(15-17,20,21)^ cognitive-linguistic functions^(17)^, behavioral skills^(15,21)^ and sensory profile^(17)^ were used.

One study assessed the level of stress^(21)^ and another checked the acceptable noise level^(17)^. Social perception and cortical auditory evoked potential were also assessed^(22)^. Thus, pre- and/or post-evaluation questionnaires were applied, but they were heterogeneous (Chart 1).

**Chart 1:** Questionnaires and protocols found in the studies

AUDITORY RECOGNITION	FUNCTIONS AUDITORY SKILLS	BEHAVIORAL	SENSORY PROFILE	OTHER PROTOCOLS
Sentence recognition test	APHAB	TRF	SSP	ANL
BKB-SIN	S.I.F.T.E.R	CBCL		Stress level
LTC-2	CHAPS	Behavioral characterization		Auditory temporal processing test
CNC	LIFE-R			Social Perception
	CHILD			CAEP

Note: BKB-SIN: *Bamford-Kowal-Bench Speech-in-Noise Test* ^(15-17)^; LTC-2: *The Listening Comprehension Test* ^(16)^; CNC: *Consonant-Núcleos-Consonant-Word* ^((20,21)^ *;* APHAB: *Abbreviated Profile of Hearing Aid Benefit* ^(20,21)^; S.I.F.T.E.R*: Supporting Success For Children With Hearing Loss* ^(15)^; CHAPS: *Children’s Auditory Performance Scale* ^(15-17)^; LIFE-R: *Listening Inventory For Education - Revised* ^(15,16)^; CHILD: *Children’s Home Inventory for Listening Difficulties* ^(15,16)^; TRF *- Teacher's Report* ^(21)^; CBCL: *Child Behavior Checklist* ^(21)^; SSP: *Short Sensory Profile* ^(17)^; ANL: *Acceptable Noise Level test* ^(17)^; CAEP: Cortical Auditory Evoked Potential^(22)^. Source: Own authorship

### Assistive Technology

Personal RMS assistive technology was used in five studies^(15-17,20,22)^ and two studies used free-field RMS (Phonak Roger Digimaster 5000)^(18,19)^. However, one study used part of the sample with a personal system (Phonak Roger Focus with Roger Inspiro-FM transmitter) and part with a free-field system (Phonak Roger Digimaster 5000)^(21)^.

The models were different between the studies, and it is noteworthy that four of them still used frequency-modulated system technology^(15,16,20,21)^. One study did not mention the RMS model used^(22)^.

### Intervention process

**Figure 2:**
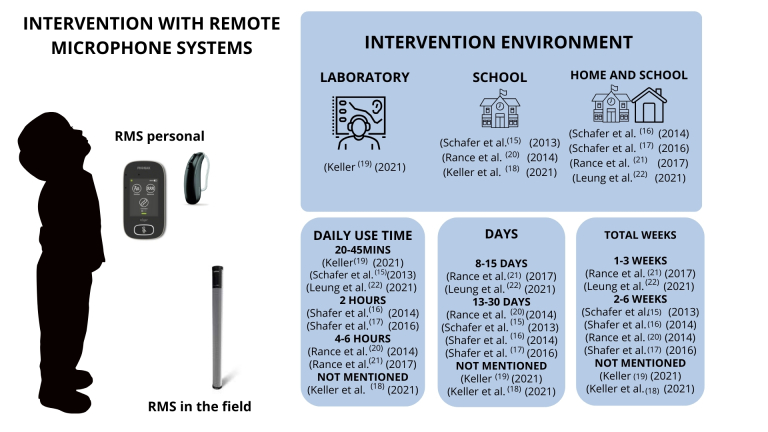
Intervention process with remote microphone systems

The intervention had different characteristics in each study, including the environment, daily time, days of intervention, and duration in weeks (Figure 2). The description of the intervention process varies between the studies, two of which alternated the use of the RMS on and off during the intervention process^(18,19)^.

In the study by Keller et al.^(18)^ there was no reference to the duration of the session. In the studies by Schafer et al. and Schafer et al.^(16,17)^, the intervention took place over 2 hours. In the studies by Rance et al.^(20,21)^ the duration of the session was between 4 and 6 hours of technology use, and in the second study, there were two 20-minute acclimatization sessions on two consecutive days.

The study by Keller^(19)^ included three conditions of use of the RMS, with 30 minutes of use per condition. In the study by Schafer et al.^(15)^, the technology was used for 45 minutes, with an acclimatization period of 15 to 20 minutes a day for a week.

Two studies did not mention the intervention time in days^(18,19)^. The study by Rance et al.^(20)^ took place on five days of the week and in the study by Rance et al.^(21)^ the intervention period was 8 to 10 days. In the study by Schafer et al.^(15)^ the intervention took place over 27 days, including periods with the RMS on (8 days), RMS off (6 days), and then RMS back on (13 days). In the study by Schafer et al.^(16)^ it was between 20 and 30 days, a period of days similar to the study by Schafer et al.^(17)^, which was an average of 30 days. In the study by Leung et al.^(22)^ the period was nine days of auditory training on a computer lasting 20 to 30 minutes, using the RMS during the sessions, and at school for 15 days (Figure 2).

With regard to the location of the intervention, seven studies included the school environment^(15-18,20-22)^, and of these, four carried out the intervention at home at the same time^(16,17,21,22)^. The only laboratory-only study which did not perform the intervention in a school environment^(19)^ applied background noise to simulate typical school noise. Two studies filmed all the intervention sessions to analyze the subjects' behavior^(18,19)^. However, in the studies included, there was no homogeneous detailing of the physical and acoustic characteristics of the environments (dimensions, flooring, furniture); two studies reported controlling background noise^(18,21)^ during the intervention - below 65 and 45 dB, respectively. The details of the pedagogical activity involving the school environment were also heterogeneous, with one study reporting that the students were seated in a circle^(18)^ and another that there was a group activity^(18)^.

### Objectives and outcomes

The objectives and outcomes were similar in all studies, as they proposed to study the possible effects of the use of RMS technology in children and adolescents with ASD in auditory aspects, as shown in Table 2.

**Table 2:** Objectives and outcomes of the scoping review studies

Author/year/place	Objective	Outcome
Keller et al.^(18)^ (2021), United States of America	To verify the effectiveness of the use of RMS in the functional listening performance of preschoolers with ASD and language disorders	Improved performance in functional listening and a reduction in response time to verbal commands.
Keller^(19)^ (2021), United States of America	To examine the effects of the use of RMS on the listening difficulties in children with ASD and severe language disorder	Improved auditory performance in minimally verbal ASD with RMS.
Rance et al.^(20)^ (2014), Australia	Evaluating mono and binaural processing skills in children with ASD	The FM system has shown clear benefits for hearing and communication in children with ASD. There was an improvement in speech perception in noise, social social interaction and classroom behavior.
Rance et al.^(21)^ (2017), Australia	To evaluate the effect of auditory intervention on the response to stress in school children with ASD.	Improved speech perception in (noisy) everyday listening conditions, with better interaction, and reduced stress related to hearing.
Schafer et al.^(15)^ (2013), United States of America	To verify the benefit of the FM System in children with ASD and ADHD through measures of performance and speech recognition in noise, and behavior observed in the classroom;	Improved speech recognition in noise and behavior during classroom tasks with the use of RMS.
Schafer et al.^(16)^ (2014), United States of America	Report on speech recognition performance in noise; listening comprehension; by participants, parents and teachers with RM	Improved speech recognition and communication
Schafer et al.^(17)^ (2016), United States of America	To evaluate the potential benefits of RM in children with ASD.	Improved speech in noise, attention, auditory memory, noise tolerance and listening comprehension
Leung et al.^(22)^ (2021), New Zealand	Exploring the effects of auditory training on the social perception skills of children with ASD	Improved behavioral performance in social perception measures, electrophysiological results showed changes in neural activity in response to post-intervention changes

Source: Own authorship

## DISCUSSION

This study analyzed and synthesized eight scientific articles, published in international literature, with the aim of mapping studies with children and adolescents with ASD that used the RM system.

The studies found are concentrated in developed countries: the USA^(15-19)^, Australia^(20,21)^, and New Zealand^(22)^, with a greater number in the USA. No Brazilian study was found. The non-inclusion of Brazilian studies in this review may be a reflection of the use and availability of RM technology in the Brazilian National Health System (SUS). Access is currently regulated for the hearing-impaired population enrolled in the school system^(23)^. As studies are scarce, there is still no national recommendation, or international guidelines, for the use of RM in this population.

It can be considered that the publications included are recent, since all the studies found in this scoping review are from the last decade, and it can be inferred that the use of RMS in subjects with ASD is still under-explored.

The studies investigated children and adolescents of different age groups. One study^(18)^ only involved young children between three and four years old, while the other studies^(15-21)^ included children from six years old to adolescents. The wider age range of the studies reflects the methodological differences found, since each age group has its own specifications for selecting assistive technologies and the intervention process, in a more global way. The selection of older children rather than pre-schoolers as participants in most of the studies in this Review may be associated with the fact that the use of personal RMS is challenging in young children - it can cause the occlusion effect, or even accidents with the olives/receivers. Another limiting factor would be the availability of RMS in the free field, as it is not suitable for reverberant environments. In this aspect of characterizing the environmental space in which the RMS intervened, the details are heterogeneous between the studies. Despite this, it is an important aspect to consider in intervention and teaching environments, since controlling noise and reverberation contributes positively to hearing accessibility.

The sensory alterations presented by children with ASD, such as hypersensitivity to sounds or acceptance of wearing devices in the ears, language and communication difficulties, or difficulties in reporting personal experiences verbally^(24,25)^, may have restricted the number of studies with children under the age of six. In at least five studies^(17,19-22)^, adolescents used RMS with an in-ear receiver and did not show any resistance to use.

Two included studies used free-field RMS^(18,19)^. In the study by Rance et al.^(21)^, participants were divided into two groups according to age. The group of younger children (6 years old) used free-field RMS, while the older children (16 years old) used RMS with an in-ear receiver, possibly due to the challenges mentioned above. Studies carried out by Schafer et al.^(3)^ and Feldman et al.^(4)^ used personal RMS in children from six years old to young adults, thus corroborating that the use in older children would be facilitated in the intervention process, as well as being more collaborative with the use of personal RMS in any intervention environment.

Confirmation of the diagnosis of ASD is fundamental for defining the therapeutic approach, as well as the indication of assistive technology. In this review, the diagnostic instrument/assessment for ASD was not clearly reported in one article^(15)^, but the authors mention that the study population came from one service, so it is possible that the diagnosis was previously established according to the criteria adopted by the institution. Even so, confirming this diagnosis at the time of the research could easily be done by applying a protocol for diagnosing ASD in the subjects of his study. A similar approach was taken in another study^(22)^, in which the CARS-2 protocol was applied to confirm the participants' diagnosis of ASD. All the other studies^(16-21)^ attested to the diagnosis of ASD through instruments or access to diagnostic reports, even when the parents and/or school reported the diagnosis. The level of support of the subjects included in the studies was not detailed, which does not allow inferences about the indication of the use of RMS in the population with ASD from this indication. The classification of the level of support in ASD was proposed in the DSM-V in 2013. As the studies included were from 2013 to 2021, it is possible that the adoption of these classification criteria was not adopted in the early studies, but that they could have added value to the more recent studies.

The participants' language development is described as verbal. However, the level of language was heterogeneous: there were children with little linguistic repertoire^(17-19)^, others with greater linguistic skills^(17,20,21)^, and even those who were verbal, without having had a language characterization^(15,16,22)^. Evaluating the verbal skills of autistic children is of great importance, as most of them have different language and speech difficulties^(26,27)^. Thus, understanding whether an individual with ASD is verbal favors the possibility of applying questionnaires aimed at the research participant regarding the use of the RMS, and can contribute to collecting information during the use of the device, as was done in the study by Shafer et al.^(17)^, in which the children answered the LIFE-R questionnaire. The possibility of measuring the results of the use of assistive technology more widely can provide important support not only in establishing criteria for the indication of RMS but also in therapeutic monitoring, favoring adjustments to the technology during its use.

With regard to the participants' hearing acuity, in one study the hearing assessment was not carried out^(20)^, and in another, only the report of guardians was taken into account^(19)^. This information is relevant and worrying, given that even mild, unidentified hearing loss can cause damage both to the development of hearing skills and to the process of language development^(28)^. The diagnosis of the presence or absence of peripheral hearing loss is fundamental since the indication of assistive technology requires this information. Thus, in the absence of prior information on hearing acuity, priority should be given to hearing screening or audiological assessment^(29)^. All the other studies^(15-17,21,22)^ confirmed the individuals' hearing through tests such as transient otoacoustic emissions (TEOAE), distortion product otoacoustic emissions (DPOAE), tonal hearing thresholds, free field measurements and/or retrospective analysis of medical records. In addition to this measurement, checking the RMS itself by measuring its transparency is essential to promote the best and most individualized adaptation possible, although its application was not mentioned in the studies included.

During the intervention process, there was variability in the time of use day/session in minutes or hours with RMS. In the studies by Schafer et al.^(16,17)^, Keller et al.^(18)^, Schafer et al.^(20,21)^ the intervention time was between 2 and 6 hours per day, while in the other studies, this time was shorter or not reported^(15,19,22)^. It is important to note that none of the eight studies reported confirmation of the use of assistive technology by teachers/parents through daily descriptive monitoring of RMS use during the intervention. This record would make it easier to monitor the use and effectiveness of the technology since it depends on use by the main speaker - in the case of the studies included, teachers and parents.

Of the eight studies, two did not mention the intervention time^(18,19)^, and six reported an intervention period of 8 to 10 days (two weeks) and a maximum of 30 days (6 weeks)^(15-17,20-22)^.

The benefits and criteria for indicating RMS for the hearing impaired (HI) population are well known and well described in the literature, a scenario not yet found for the ASD population. In the studies by Benítez-Barrera et al.^(5,6,30)^, RMS intervention was carried out on children with AD over two weekends, alternating between RMS and no RMS. In the two studies on RMS at home by children with AD, and the studies on RMS and ASD presented here, from 8 to 30 days of intervention, it was possible to observe benefits from assistive technology, with improvements in speech perception in all the studies. Even so, looking at the impact of improved auditory accessibility and understanding the differences between AD and ASD, we can infer that the longer intervention time benefits auditory input through RMS technology, as the improvement in speech perception over time favors the auditory plasticity mechanism^(31)^.

With regard to the environment, only one study carried out the intervention exclusively in a laboratory with typical school noise^(19)^. The school was the main environment chosen by the researchers^(15,18,20)^ for the intervention process, although with limitations in the description of the teaching activities carried out during the observation period - a context that can also interfere with background noise. In this sense, two studies refer to the control of background noise^(18,21)^, however, the detailing of the characteristics of the environment in physical and acoustic terms is limited and there is no standard of description.

The intervention took place at home and/or at school in four studies^(16,17,21,22)^. The use of RMS in the home environment is still understudied, probably due to the lack of evidence in favor of its feasibility and effectiveness in the home environment^(5)^, which has more variables and researchers have less control over them. However, as it is an environment in which children spend a lot of time during the week, the school, as well as being an environment of interaction and where there are environmental distractors^(32)^, becomes the main environment for intervention and observation of the benefit of the use of RMS, with instruments that allow this benefit to be monitored.

Moreover, teachers must be trained in new strategies that will facilitate the children's learning process^(33)^. In the study by Sposito et al.^(34)^, the lack of support from teachers was cited as a challenge by children and adolescents with AD for the use of RMS in the classroom.

Different instruments were used for the pre- and post-use of RMS in the children. The ANL test was used in one study^(17)^ to observe the noise level that was acceptable with or without the use of RMS. The study by Koiek et al.^(35)^ also applied the ANL to 93 children of both sexes, aged 7-12, separated into two groups, with and without a learning disorder, and concluded that the group with ASD tolerated less background noise. In the study by Freyaldenhoven and Smiley^(36)^, the acceptable noise level was assessed in 32 children aged 8-12 with normal hearing. They assessed the comfortable hearing level (MCLs) and the maximum background noise levels (BNLs) to obtain the ANL and found that the acceptable noise level was independent of the gender and age of the children in the study. The results suggest research with children with hearing loss and ANL to predict the success or rejection of hearing aids. These studies also reported that the test applies to children quickly^(35,36)^. The CNC test was only used in the two studies by Rance et al.^(20,21)^. This is an old test^(37)^ for assessing speech perception in subjects with or without hearing difficulties^(38-40)^.

Auditory temporal processing skills were assessed using the technique of Álcantara et al.^(41)^, who observed a difference in the children with ASD in their study when compared to controls (matched by age and Intelligence Quotient), suggesting that children with ASD had reduced sensitivity to temporal modulation. Rance et al.^(20)^ used this technique, showing that younger children with ASD were less sensitive to temporal changes. The LISN-S test, which assesses binaural processing, was only applied by Rance et al.^(20)^, who observed poor integration in children with ASD. The test (LISN-S) was also reported in the study by Cameron et al.^(42)^ who evaluated ten children at risk of Central Auditory Processing Disorder (CAPD), with worse results in all LISN-S measures for these children than for their age-matched controls, concluding that this test would be promising in the CAP test battery.

In these three studies, Schafer et al.^(15-17)^ used the BKB-SIN in common to evaluate speech recognition in noise^(43)^. The BKB-SIN was applied in the study by Ng et al.^(44)^ in mixed groups composed of adults without AD, children without AD, and children with AD, demonstrating the reliability of the results. Other studies with children have also used this test in their investigations^(45-49)^.

The LTC-2 test assesses auditory comprehension and was carried out in the study by Schafer et al.^(16)^.

The studies by Keller et al.^(18)^ and Keller^(19)^ did not use standardized instruments to assess speech recognition. The authors adopted an observational procedure of listening performance through questions to the children, based on three categories, and following the level of communication. Although they did not use a validated instrument, they were able to prove an improvement in speech recognition in these minimally verbal children. The use of validated instruments is important because they present standards of normality that favor the interpretation of results clearly. Although the included studies evaluated different skills, there was an improvement in speech perception in all the subjects with ASD who used the RMS.

Among the questionnaires that were applied, CHAPS was used in three of the studies in this review^(15-17)^. LIFE was also chosen and applied^(16,17,20)^, APHAB was used in two studies^(20,21)^ and, finally, CHILD was applied in two studies^(16,17)^. The purpose of these questionnaires is to extract information about listening skills and difficulties in the school environment and/or at home. These instruments have also been used in studies with subjects with APD and the use of RMS, as hearing screening, in elderly people with AD and use of hearing aids, as well as in subjects with ASD^(50-55)^. The selection of the instrument to gather information on listening skills and difficulties is particularly relevant in the population with non-verbal ASD, and structured observation is an important marker for this audience.

Behavioral aspects were assessed with the CBCL and TRF in the study by Rance et al.^(21)^ and a behavioral characterization in the study by Schafer et al.^(15)^. The CBCL helps to identify emotional disorders such as depression and anxiety, attention difficulties, as well as aggressive behavior, and there has been research into children in custody, anxiety and affective problems, and bipolar disorder. TRF identifies children's interfering behaviors in the school environment^(56-59)^. Improved hearing accessibility can also change the type and frequency of interfering behaviors, which is yet another variable to be observed.

The sensory profile was only explored in one study^(17)^, where they observed that participants with ASD when using the RMS, had less difficulty in auditory filtering and visual/auditory sensitivity. In the study by Lyons-Warren et al.^(60)^, when characterizing these children with ASD using the SSP instrument, they observed among the groups that there were children who had isolated hearing alterations and another group with concomitant differences in hearing and taste. The study by O'Brien et al.^(61)^ corroborates these findings in children with ASD, as they found high levels of auditory/visual hypersensitivity compared to controls. Thus, the SSP is an instrument widely used in this population^(62,63)^, and the study by Schafer et al.^(17)^ in this review, when comparing with and without RMS, showed an improvement in the hearing condition with the use of RMS in this population.

The level of stress was studied by checking the concentration of salivary cortisol in the children, which is considered an important biomarker of stress^(64,65)^. Studies such as those by Tordjman et al.^(66)^ and Ogawa et al.^(67)^ have been carried out with children with ASD to assess cortisol levels, as these children show variable diurnal regulation and greater reactivity, especially in older children^(68)^. Only one study in this review, that of Rance et al.^(21)^, studied stress through this biomarker, and it was observed that cortisol levels were high in children who had worse speech perception, generating greater auditory effort, which would probably be associated with a higher level of anxiety when performing auditory tasks.

The ACS was used in one of the studies^(22)^, which is a measure of social perception^(69)^ and is a scale that can be carried out individually or in groups. Leung et al.^(22)^ found that when comparing the use of RMS and auditory training on the computer for three weeks, there was a significant improvement in social perception ability, so much so that children with ASD outperformed the control group (CG, which received no intervention) in the category of naming affect and matched the prosody scores of social perception with the peers of CG children. These results allow us to infer that the intervention process was beneficial for children with ASD and that it favored an improvement in social perception.

In the study by Leung et al.^(22)^, the authors carried out the electrophysiological measure of Mismatch response (MMR) in children with ASD, and verified changes in the post-intervention time windows, although the results showed that it was not within the expected “normal” range for auditory processing. Thus, it may be thought that the intervention time was insufficient to normalize this processing, but it has already brought benefits to the children in the study when compared with their performance in the pre-intervention stage, and when compared with the post-intervention CG. Even so, studies need to be carried out to corroborate these findings with better control conditions, as the MMR is promising for investigating the auditory maturation of children, as mentioned in the systematic review and meta-analysis carried out by Themas et al.^(70)^. In addition, MMR can be related bilaterally in the auditory and frontal cortex, with laterality to the left hemisphere when speech is processed^(71)^. Other electrophysiological measures can also be used as markers of changes in auditory skills, such as those included in the auditory processing assessment battery^(72)^.

The studies^(15-22)^ presented similarities in their objectives, that is, the process of intervention with RMS in the population with autism was the baseline and the outcome of the observation and characterization of the pre- and post-intervention findings. There was also a similarity in the results, which showed that improved speech perception was the main benefit of the RMS intervention. However, the studies listed here indicated other possible benefits that assistive technology could promote, such as behavioral and social aspects, reduction in stress levels, modification of the electrophysiological response after use of RMS and auditory training, and minimization of auditory effort.

## CONCLUSION

The results of the scoping review showed that children and adolescents with ASD benefited from the RMS assistive technology intervention process by improving speech perception. These results have contributed to the development process of these children in school, family, and social environments.

Even so, more research should be carried out with a larger sample size and similar methodologies to provide evidence regarding the criteria for indicating the technology. The use of these tools to more effectively measure the impact of the auditory input favored by the use of RMS can highlight not only the positive results regarding speech perception, but also secondary impacts, which are already evident in the studies in this review, but which require further studies to corroborate these findings.

Hence, RMS is promising for use in this population, as it minimizes difficulties in speech perception and provides secondary behavioral benefits, reducing listening effort and stress.

## Appendix 1 Database search strategy

**Table:**

Database	Search (16th March 2023)
**Embase**	("Autism Spectrum Disorder" OR "Autism Spectrum Disorders" OR "Autistic Spectrum Disorder" OR "Autistic Spectrum Disorders" OR "Autistic Disorder" OR "Infantile Autism" OR "Autism" OR "Early Infantile Autism") AND ( "Sensory Aids" OR "Sensory Aid" OR "Self-Help Devices" OR "Self Help Devices" OR "Remote Microphone Systems" OR "Microphone System" OR "remote microphone technology" OR "Remote Microphone System" OR "FM system" OR "frequency modulation system")
**LILACS**	("Tecnologia Assistiva" OR "Dispositivos de Autoayuda" OR "Dispositivos Asistivos" OR "Dispositivos de Autoajuda" OR "Equipamentos Assistivos" OR "Equipamentos de Autoajuda" OR "Sistema de frequência modulada" OR "Sistema FM" OR "Microfone Remoto" OR "Assistive Technology" OR "Self-Help Devices" OR "Remote Microphone Systems" OR "Microphone System" OR "remote microphone technology" OR "Remote Microphone System" OR "FM system" OR "frequency modulation system" OR "remoto de micrófono" OR "sistema de frecuencia modulada" OR "tecnología de asistencia") AND (("Transtorno Autístico" OR "Autismo" OR "Autismo Infantil" OR "Síndrome de Kanner" OR "Transtorno do Espectro Autista" OR "Autism Spectrum Disorder" OR "Kanner's Syndrome" OR "Infantile Autism" OR "Autistic Disorder" OR "Transtorno del Espectro Autista"))
**PubMed**	("Autism Spectrum Disorder"[Mesh Terms] OR "Autism Spectrum Disorder" OR "Autism Spectrum Disorders" OR "Autistic Spectrum Disorder" OR "Autistic Spectrum Disorders" OR "Autistic Disorder" [Mesh Terms] OR "Autistic Disorder" OR "Kanner's Syndrome" OR "Kanner Syndrome" OR "Infantile Autism" OR "Autism" OR "Early Infantile Autism") AND ("Sensory Aids" [MeSH Terms] OR "Sensory Aids" OR "Sensory Aid" OR "Self-Help Devices" [MeSH Terms] OR "Self-Help Devices" OR "Self Help Devices" OR "Self-Help Device" OR "Remote Microphone Systems" OR "Microphone System" OR "remote microphone technology" OR "Remote Microphone System" OR "FM system" OR "frequency modulation system")
**Scopus**	("Autism Spectrum Disorder" OR "Autism Spectrum Disorders" OR "Autistic Spectrum Disorder" OR "Autistic Spectrum Disorders" OR "Autistic Disorder" OR "Autistic Disorder" OR "Kanner's Syndrome" OR "Kanner Syndrome" OR "Infantile Autism" OR "Autism" OR "Early Infantile Autism") AND ("Sensory Aids" OR "Sensory Aid" OR "Self-Help Devices" OR "Self Help Devices" OR "Self-Help Device" OR "Remote Microphone Systems" OR "Microphone System" OR "remote microphone technology" OR "Remote Microphone System" OR "FM system" OR "frequency modulation system")
**Web of Science**	("Autism Spectrum Disorder" OR "Autism Spectrum Disorders" OR "Autistic Spectrum Disorder" OR "Autistic Spectrum Disorders" OR "Autistic Disorder" OR "Kanner's Syndrome" OR "Kanner Syndrome" OR "Infantile Autism" OR "Autism" OR "Early Infantile Autism") AND ("Sensory Aids" OR "Sensory Aids" OR "Sensory Aid" OR "Self Help Devices" OR "Self-Help Device" OR "Remote Microphone Systems" OR "Microphone System" OR "remote microphone technology" OR "Remote Microphone System" OR "FM system" OR "frequency modulation system")
**Google Scholar**	("Autism" OR"Autism Spectrum Disorder" OR "Autistic Disorder") AND (“remote microphone system” OR “frequency modulation system” OR "sensory aids")
**ProQuest**	("Autism Spectrum Disorder" OR "Autism Spectrum Disorder" OR "Autism Spectrum Disorders" OR "Autistic Spectrum Disorder" OR "Autistic Spectrum Disorders" OR "Autistic Disorder" OR "Autistic Disorder" OR "Kanner's Syndrome" OR "Kanner Syndrome" OR "Infantile Autism" OR "Autism" OR "Early Infantile Autism") AND ("Sensory Aids" OR "Sensory Aids" OR "Sensory Aid" OR "Self-Help Devices" OR "Self-Help Devices" OR "Self Help Devices" OR "Self-Help Device" OR "Remote Microphone Systems" OR "Microphone System" OR "remote microphone technology" OR "Remote Microphone System" OR "FM system" OR "frequency modulation system")

## Appendix 2 Reason for exclusion criteria

**Table:**

Author, Year	Reason for exclusion
Dunn A, James P, Pelosi A, Sorensen E, Oleson J; 2021	**1**
Feldman JI, Thompson E, Davis H, Keceli-Kaysili B, Dunham K, Woynaroski T. et al.; 2022	**2**
Hess KL, Morrier MJ, Heflin, Heflin LJ, Ivey ML; 2008	**3**
Rance G; 2013	**3**
Schafer, E C; Gopal, K V; Mathews, L; Thompson, S; Kaiser, K; McCullough, S; Jones, J; Castillo, P; Canale, E; Hutcheson, A; 2019	**2**
Teleaudiology Today, 2022	**3**
Westby, C, 2014	**3**
1) Non-intervention studies; 2) Studies with adults/elderly people; 3) reviews, opinion articles, posters, letters, and conference abstracts.	
**References**	
1. Dunn A, James P, Pelosi A, Sorensen E, Oleson J. Managing listening difficulties in patients with ASD and normal hearing sensitivity. Hearing Review. 2021.	
2. Feldman JI, Thompson E, Davis H, Keceli-Kaysili B, Dunham K, Woynaroski T et al. Remote microphone systems can improve listening-in-noise accuracy and listening effort for youth with autism. Ear Hear. 2022;43^(2)^:436-447.	
3. Hess KL, Morrier MJ, Heflin, Heflin LJ, Ivey ML. Autism treatment survey: services received by children with autism spectrum disorders in public school classrooms. J Autism Dev Disord. 2008;38:961-971.	
4. Rance G. In autism study, FM system enhances listening, attention, and behavior. Journal club. 2013;66(7)4-5.	
5. Schafer EC, Gopa KV, Mathews L, Thompson S, Kaiser K, McCullough S et al. Effects of auditory training and remote microphone technology on the behavioral performance of children and young adults who have autism spectrum disorder. J Am Acad Audiol. 2019;30:431-443.	
6. Teleaudiology today [internet].Can Remote Microphone Systems Improve Listening-in-noise Accuracy in Children with Autism? The Hearing Journal. 2022*;*75^(3)^:10. [cited 2023 nov 15]. available from: https://journals.lww.com/thehearingjournal/Fulltext/2022/03000/Can_Remote_Microphone_Systems_Improve.7.aspx	
7. Westby, C. FM; 2014. Systems with students with autism spectrum disorders or attention deficit hyperactivity disorder. [cited 2023 nov 15] [about 1 screens] Available from: https://journals.sagepub.com/doi/10.1177/1048395014527568b.	
