# Supplementary material for: Remote microphone systems in children and adolescents with autism spectrum disorders: a scoping review
Source: Codas. 2025 Feb 24;37(2):e20230310. doi: 10.1590/2317-1782/e20230310en (PMC11864769; doi:10.1590/2317-1782/e20230310en)
Supplement: Translation

# Translation: Sistemas de microfone remoto em crianças e adolescentes com transtorno do espectro autista: revisão de escopo

**DOI:** 10.1590/2317-1782/e20230310pt

**Authors:** Bianca Stephany Barbosa Vital, Karen Melissa Gonzaga dos Santos, Aryelly Dayane da Silva Nunes Araújo, Joseli Soares Brazorotto, Regina Tangerino de Souza Jacob, Karinna Veríssimo Meira Taveira, Sheila Andreoli Balen

**Affiliations:** 1 Programa Associado de Pós-graduação em Fonoaudiologia, Universidade Federal do Rio Grande do Norte – UFRN - Natal (RN), Brasil.; 2 Universidade Federal da Paraíba – UFPB - João Pessoa (PB), Brasil,; 3 Universidade Estadual de Ciências da Saúde de Alagoas – UNCISAL - Maceió (AL), Brasil.; 4 Laboratório de Inovação Tecnológica em Saúde – LAIS, Universidade Federal do Rio Grande do Norte – UFRN - Natal (RN), Brasil.; 5 Departamento de Fonoaudiologia, Universidade Federal do Rio Grande do Norte – UFRN - Natal (RN), Brasil.; 6 Departamento de Fonoaudiologia, Faculdade de Odontologia de Bauru – FOB, Universidade de São Paulo – USP - Bauru (SP), Brasil.; 7 Departamento de Morfologia, Universidade Federal do Rio Grande do Norte – UFRN - Natal (RN), Brasil.

**Keywords:** Transtorno do Espectro Autista, Microfone Remoto, Percepção Auditiva, Crianças, Adolescentes

## Abstract

**Objetivo:**

Mapear a literatura acerca do uso do Sistema de Microfone Remoto (SMR) em crianças e adolescentes com Transtorno do Espectro Autista (TEA).

**Método:**

Revisão de Escopo com recomendações do Instituto Joanna Briggs e do checklist PRISMA-ScR. Foi realizada busca nas bases de dados: Pubmed, Embase, Scopus, Web of Science, Lilacs e na literatura cinzenta Google Scholar e ProQuest, além de listas de referências dos estudos incluídos e consulta a experts. Foram incluídos estudos de intervenção, com crianças e adolescentes com TEA que fizeram uso do SMR, sem restrição de gênero, idioma, idade, tempo de publicação, etnia ou localização geográfica.

**Resultados:**

Foram identificados 709 estudos na fase 1. Após a leitura de 14 textos completos com elegibilidade, oito estudos foram elegíveis. Os estudos foram heterogêneos quanto ao modelo do SMR (individual ou em campo), dos testes aplicados, período e local de intervenção. Constatou-se favorecimento da percepção de fala com melhora na interação social, comportamento, atenção e memória auditiva, tolerância ao ruído e redução do estresse, além de modificação na atividade neural a partir da avaliação eletrofisiológica.

**Conclusão:**

O uso do SMR apresentou benefícios na percepção de fala, interação social e comportamento de adolescentes e crianças com TEA. Há necessidade de mais estudos para definir protocolos e parâmetros de indicação nesta população.

## INTRODUÇÃO

Nas salas de aula ocorrem ruídos ambientais das mais diversas formas, como as conversas entre os alunos, a movimentação de cadeiras e mesas, o amassar das folhas de caderno, os sons do ventilador e do ar-condicionado, do corredor e da rua, pela janela, dentre tantos outros ruídos que podem dificultar o acesso à fala do professor^(1)^.

Inseridas no contexto escolar encontram-se crianças com Transtorno do Espectro Autista (TEA). O TEA é um transtorno do neurodesenvolvimento com crescente prevalência e que ocorre de forma heterogênea, sendo caracterizado pelas dificuldades na comunicação social, comportamentos repetitivos, foco de interesse e alterações sensoriais^(2)^.

Estas crianças podem apresentar dificuldades persistentes na comunicação e na interação social, como também padrões de comportamentos restritos e repetitivos (sejam comportamentais ou de interesses/atividades). Intervenções para que estas crianças e adolescentes desenvolvam suas habilidades e potencialidades, seja no ambiente escolar, familiar ou social são fundamentais^(2)^.

Pesquisas estão sendo realizadas em escolares com TEA e o uso do sistema de microfone remoto (SMR) por apresentarem “*listening difficulties*”. Estudos^(3,4)^ demonstraram potenciais benefícios quando realizada intervenção com sistema de microfone remoto em crianças e jovens adultos^(3,4)^ com benefício na percepção de fala no ambiente escolar pós intervenção. O SMR é uma tecnologia assistiva amplamente estudada e indicada em outras populações, principalmente para indivíduos com deficiência auditiva (DA)^(5-7)^ e com transtorno do processamento auditivo central^(8-10)^.

O termo SMR compreende tecnologias que apresentam como forma de transmissão o sinal digital e a frequência modulada (FM)^(11)^, e seu uso pode ser pessoal ou em campo. O primeiro é um aparelho composto por um transmissor que capta o sinal acústico do falante principal (professor) e transmite por sinal digital para o ouvinte (aluno) que está fazendo uso de um receptor na orelha. Este sinal acústico chega ao ouvinte com redução do ruído e com melhor sinal acústico, sendo este de uso individual^(11)^.

Já no SMR em campo, o intuito é o uso coletivo, onde a voz do falante principal é amplificada e transmitida por uma caixa de som em campo para um maior número de pessoas, como os estudantes de uma sala de aula^(12)^. A Sociedade de Audiologia da Austrália^(13)^ ressaltou a importância dos audiologistas na intervenção de crianças com TEA recomendando o SMR em diversos ambientes.

Diante deste panorama, o objetivo dessa revisão de escopo foi mapear os estudos sobre o uso do SMR em crianças e adolescentes com TEA.

## MÉTODO

### Protocolo e Registro

Este estudo trata de uma revisão de escopo seguindo as diretrizes metodológicas do Instituto *Joanna Briggs* (JBI) e recomendações do checklist PRISMA-ScR (*Preferred Report items for systematic reviews and Meta-analyses extension of Scoping Reviews, 2018*). Esse tipo de estudo utiliza uma abordagem sistemática para promover uma síntese do conhecimento, identificando os principais conceitos, teorias, fontes e lacunas do conhecimento^(14)^. O protocolo de pesquisa do presente estudo foi submetido para avaliação e registro na plataforma Open Science Framework (OSF), DOI: 10.17605/OSF.IO/Q4GBF.

### Critério de Elegibilidade

Utilizou-se o acrônimo “PCC”, para elencar os estudos elegíveis para esta revisão: P = População (crianças e adolescentes com diagnóstico de TEA); C = Conceito (Evidências pós intervenção com Microfone Remoto em crianças e adolescentes com TEA); C = Contexto (uso do SMR).

### Critérios de Inclusão

Estudos que avaliam o uso do SMR em crianças e adolescentes com TEA sem restrição a gênero, idioma, idade, tempo de publicação, etnia ou localização geográfica.

### Critérios de exclusão

Os seguintes critérios de exclusão foram realizados: participantes com perda auditiva, estudos sem pré e/ou pós intervenção com SMR; estudos com outros desenhos que não de intervenção; estudos com crianças e adolescentes sem diagnóstico de TEA; estudos com adultos e/ou idosos; tipos de publicações como revisões sistemáticas, meta-análises, livros, *guidelines*, *websites*, blogs.

### Busca na literatura

A estratégia de busca foi realizada, primeiramente no Pubmed, sendo adaptada para as demais bases utilizadas nesta revisão: Embase, Scopus, Web of Science, Lilacs, Google Scholar e ProQuest, no dia 16 de março de 2023 (Apêndice 1).

Após a busca em cada base de dados foi feita a exportação dos resultados para o *Software Mendeley*, quando foram identificados os artigos duplicados. A partir deste ponto o arquivo foi, então, salvo e exportado para o *website Rayyan* (https://www.rayyan.ai/), onde foi realizada novamente checagem de duplicados e início da leitura de título e resumo por dois revisores independentes.

### Seleção dos estudos

A seleção dos estudos ocorreu em duas fases. Na primeira fase dois revisores analisaram de forma independente o título e resumo a partir dos critérios de elegibilidade. Essa etapa foi realizada no *website Rayyan*. Na segunda fase, foi realizada a leitura dos textos completos pelos mesmos revisores de forma independente. Nas duas fases foi realizada reunião de consenso antes de finalizar a etapa. Quando não houve consenso entre os R1 e R2 quanto a inclusão/exclusão de um ou mais estudos, o terceiro revisor fez a análise para tomada de decisão (Apêndice 2).

As listas de referências dos estudos incluídos na fase 2 foram analisadas, assim como foram consultados três *experts* da área, para identificação de estudos que possam não ter sido recuperados na busca inicial. Estes experts foram contactados por serem pesquisadores doutores com estudos publicados na temática apresentada nesta revisão provenientes de países diferentes como Austrália, Nova Zelândia e Estados Unidos. Estes experts eram autores de estudos já identificados na busca na base de dados, sendo que dos quatro artigos indicados por eles somente um foi incluído, visto que dois eram duplicados e um não tinha os critérios de desenho do estudo conforme pode ser visualizado na Figura 1.

**Figura 1:** Diagrama de fluxo conforme diretrizes do PRISMA-ScR (adaptado)

Fonte: Adaptação Prisma-ScR pela autora

### Análise e extração dos dados

Foram extraídos os dados do estudo como autor, ano de publicação, país, objetivo, amostra, idade, instrumento de diagnóstico do TEA, nível de linguagem, acuidade audiológica pré intervenção, questionários/protocolos (pré e pós intervenção), tecnologia assistiva, processo de intervenção e principais desfechos.

Todos os dados relacionados à revisão de escopo foram extraídos e mapeados, sendo realizada uma síntese qualitativa.

## RESULTADOS

### Seleção dos estudos

A seleção dos estudos ocorreu conforme apresentado na Figura 1. Um total de 709 estudos foram recuperados nas bases eletrônicas, sendo 557 da base de dados e 152 da literatura cinzenta. Após inserção no software Mendeley permaneceram 585 estudos após serem retiradas as duplicatas que seguiram para análise do título e resumo no software Rayyan. Seguindo os critérios de elegibilidade, 571 artigos foram excluídos, seguindo 14 e um foi inserido por indicação de expert, seguindo ao todo 15 artigos para a leitura completa na fase 2. Destes, 7 estudos foram excluídos e 8 foram incluídos na síntese qualitativa e mapeamento dos resultados (Figura 1).

### Características dos estudos e da amostra

Os estudos incluídos foram publicados entre 2013 e 2021, cinco deles nos Estados Unidos da América^(15-19)^, dois na Austrália^(20,21)^ e um na Nova Zelândia^(22)^ os grupos de pesquisa observam-se quatro vertentes com estudos de Schafer et al.^(15-17)^, Rance et al.^(20,21)^, Keller et al.^(18)^ , Keller^(19)^ e Leung et al.^(22)^.

Entre os oito estudos incluídos, seis^(15-19,22)^ realizaram intervenção com número amostral entre 8 e 14 sujeitos. Os estudos do grupo da Austrália^(20,21)^ apresentaram o maior número amostral - 20 e 26 participantes (Tabela 1). E em seis estudos, foram relatadas comorbidades associadas ao TEA^(15,16,18,19,21,22)^.

**Tabela 1:** Caracterização da amostra, confirmação do diagnóstico de TEA, modalidade de linguagem expressiva e acuidade audiológica

Autor/ano/local	Amostra (n)	Idade (anos)	Diagnóstico de TEA	Status da Linguagem	Status Audiológico
Keller et al.^(18)^ (2021), Estados Unidos da América	8	3-4	Pesquisadores e/ou equipe multidisciplinar	Verbal	Audição normal
Keller^(19)^ (2021), Estados Unidos da América	14	4-16	Pesquisadores ou equipe multidisciplinar	Verbal	Audição normal
Rance et al.^(20)^ (2014), Austrália	20	8-15	Pesquisadores e/ou equipe multidisciplinar	Verbal	Audição normal
Rance et al.^(21)^ (2017), Austrália	26	6-16	Equipe multidisciplinar	Verbal	Audição normal
Schafer et al.^(15)^ (2013), Estados Unidos da América	7	9-11	Pais e confirmado pela administração da escola	Verbal	Audição normal
Schafer et al.^(16)^ (2014), Estados Unidos da América	4	9-10	Informado pelos pais e confirmado pelos profissionais especializados	Verbal	Audição normal
Schafer et al.^(17)^ (2016), Estados Unidos da América	12	6-17	Realizado por profissionais e relatado pelos pais	Verbal	Audição normal
Leung et al.^(22)^ (2021), Nova Zelândia	12	7-13	Pesquisadores ou equipe multidisciplinar	Verbal	Audição normal

Fonte: Próprio autor

Quanto à faixa-etária dos participantes com TEA nos estudos, apenas um^(18)^ envolveu pré-escolares entre 3 e 4 anos. Dentre os demais estudos, três foram realizados com crianças maiores, com faixa-etária entre de 7 a 13 anos^(15,16,22)^, e em quatro estudos a intervenção foi realizada em crianças e adolescentes com idades de 4 a 17 anos^(17,19-21)^. Apenas um estudo^(21)^ apresentou dois grupos divididos por idade, sendo um com crianças de 6 a 12 anos e um dos 13 a 16 anos.

Foi constatado como critério de inclusão para realização da intervenção o diagnóstico de TEA ratificado. Em quatro estudos^(18-22)^ o diagnóstico foi confirmado pelos pesquisadores ou equipe multidisciplinar através de instrumentos como ADOS-2, *Autism Diagnostic Interview, Childhood Autism Rating Scale,* ASEBA, CARS-2 em que eles tinham acesso aos relatórios das avaliações.

Em três estudos a confirmação do diagnóstico foi atestada através de relato de pais e/ou escola (que disponibilizaram os relatórios)^(15-17)^, e em um estudo^(15)^ os relatórios não foram disponibilizados aos pesquisadores.

O desenvolvimento linguístico dos sujeitos não foi caracterizado pelos autores, havendo heterogeneidade nas características linguísticas referidas destas crianças e adolescentes, contudo ficou claro que todos os sujeitos eram verbais. Dois estudos^(18,19)^ usaram o termo “autismo minimamente verbal” (MVA) para descrever a linguagem de seus participantes que tinham palavras únicas e ecolalias. No primeiro estudo foi realizada avaliação prévia com protocolo *Mullen Scales of Early Learning* (MSEL) para identificação de atrasos/dificuldades na linguagem, e o segundo estudo definiu a classificação pelo módulo 1 do teste *Autism Diagnostic Observation Schedule* (ADOS-2).

Em outro estudo^(17)^ os participantes foram classificados com habilidades verbais após serem categorizados pelos examinadores do estudo em cinco categorias, sendo elas^(1)^ ecolalias e poucas palavras espontâneas^(2)^, uma a três palavras espontâneas^(3)^, produz sentença com quatro ou mais palavras^(4)^, produz duas ou três frases e^(5)^ são conversacionais. Nos estudos de Rance et al.^(20,21)^ todos os participantes da intervenção podiam falar, entender e seguir instruções verbais. Nos estudos de Schafer et al.^(15,16)^ foi possível inferir que as crianças eram verbais, a partir da aplicação do teste BKB-SIN para avaliar a habilidade de reconhecimento de fala no ruído. No estudo de Leung et al.^(22)^ todos os sujeitos apresentavam autismo de alto funcionamento, o que infere que as crianças eram verbais.

A acuidade auditiva foi previamente avaliada nos participantes (Tabela 1). Apenas um estudo^(20)^ não referiu avaliação, mas deixou implícito que os participantes tinham acuidade auditiva dentro dos padrões de normalidade. Um estudo considerou como audição normal o relato dos responsáveis ou registros médicos^(19)^; outro referiu análise retrospectiva de prontuário sobre a acuidade auditiva de ao menos uma das orelhas, destacando que foram realizadas medidas em campo livre apenas aos participantes que não aceitaram o uso de fones de ouvido^(18)^.

Os estudos de Shafer et al.^(15-17)^ e de Rance et al.^(21)^ apresentaram como critério de inclusão em seus estudos: a realização da triagem auditiva nos seis meses anteriores (nas frequências de 1, 2, 4 kHz em até 25 dB); avaliação auditiva dentro dos padrões de normalidade (250 a 6000 Hz com limiares inferiores a 20dB ou presença das Emissões Otoacústicas Evocadas Transientes - EOAT); avaliação auditiva de 250 a 8000 kHz ou presença das Emissões Otoacústicas Evocadas por Produto de Distorção (EOAPD); e a pesquisa de limiar em 250 a 4000 kHz respectivamente, para confirmar que as crianças e adolescentes dos estudos não apresentavam alteração auditiva. No estudo de Leung et al.^(22)^ a audição dos participantes foi avaliada com triagem auditiva com EOAPD.

### Questionários e protocolos pré e pós intervenção

Todos os autores utilizaram instrumentos pré e pós intervenção para avaliar o processo da intervenção com SMR. Foi observada variedade de funções/habilidades avaliadas, em consequência dos diferentes instrumentos aplicados. Foram aplicados protocolos de reconhecimento auditivo^(15-21)^, questionários para avaliar: funções/habilidades auditivas^(15-17,20,21)^ funções cognitivas-linguísticas^(17)^, habilidades comportamentais^(15,21)^ e perfil sensorial^(17)^.

Houve um estudo que avaliou o nível de estresse^(21)^, e outro que verificou o nível de ruído aceitável^(17)^. Também foi avaliada a percepção social e o potencial evocado auditivo cortical^(22)^. Desta forma, questionários pré e/ou pós avaliação foram aplicados, mas foram heterogêneos (Quadro 1).

**Quadro 1:** Questionários e protocolos encontrados nos estudos

RECONHECIMENTO AUDITIVO	FUNÇÕES HABILIDADES AUDITIVAS	COMPORTAMENTAIS	PERFIL SENSORIAL	OUTROS PROTOCOLOS
Teste de reconhecimento de frases	APHAB	TRF	SSP	ANL
BKB-SIN	S.I.F.T.E.R	CBCL		Nível de estresse
LTC-2	CHAPS	Caracterização comportamental		Teste de processamento auditivo temporal
CNC	LIFE-R			Percepção Social
	CHILD			PEAC

Legendas: BKB-SIN: *Bamford-Kowal-Bench Speech-in-Noise Test* ^(15-17)^ *;* LTC-2: *The Listening Comprehension Test* ^(16)^ *;* CNC: *Consonant-Núcleos-Consonant-Word* ^(20,21)^ *;* APHAB: *Abbreviated Profile of Hearing Aid Benefit* ^(20,21)^; S.I.F.T.E.R*: Supporting Success For Children With Hearing Loss* ^(15)^ *;* CHAPS: *Children’s Auditory Performance Scale* ^(15-17)^ *;* LIFE-R: *Listening Inventory For Education - Revised* ^(15,16)^; CHILD: *Children’s Home Inventory for Listening Difficulties* ^(15,16)^ *;* TRF *- Teacher's Report* (Formulário de Relatório não Referido)^(21)^ *;* CBCL: *Child Behavior Checklist* ^(21)^ *;* SSP: *Short Sensory Profile* ^(17)^; ANL: *Acceptable Noise Level test* ^(17)^ *;* PEAC*:* Potencial Evocado Auditivo Cortical^(22)^. Fonte: Próprio Autor

### Tecnologia Assistiva

A tecnologia assistiva de SMR pessoal foi usada em cinco estudos^(15-17,20,22)^ e dois estudos utilizaram o SMR em campo livre (Phonak Roger Digimaster 5000)^(18,19)^. Entretanto, um estudo utilizou parte da amostra com sistema pessoal (Phonak Roger Focus com transmissor Roger Inspiro-FM) e parte com sistema em campo livre Phonak Roger Digimaster 5000^(21)^.

Os modelos foram diferentes entre os estudos, e destacam-se que quatro deles ainda utilizaram a tecnologia de sistema de frequência modulada^(15,16,20,21)^. Um estudo não referiu o modelo do SMR usado^(22)^.

### Processo de intervenção

A intervenção apresentou características distintas em cada estudo, seja o ambiente, tempo diário, dias de intervenção e duração em semanas (Figura 2). A descrição do processo de intervenção é variável entre os estudos, sendo que dois deles alternaram o uso do SMR ligado e desligado no processo interventivo^(18,19)^.

**Figura 2:** Processo de intervenção com sistemas de microfone remoto

No estudo de Keller et al.^(18)^ não houve referência quanto à duração da sessão. Nos estudos de Schafer et al e Schafer et al.^(16,17)^ a intervenção ocorria durante 2 horas. Nos estudos de Rance et al^(20,21)^ a duração da sessão foi de 4 a 6 horas de uso da tecnologia, havendo no segundo estudo duas sessões de aclimatização de 20 minutos por dois dias consecutivos.

O estudo de Keller^(19)^ incluiu três condições de uso do SMR, com 30 minutos de uso por condição. No estudo de Schafer et al.^(15)^ o uso da tecnologia foi de 45 minutos, com período de aclimatização de 15 a 20 minutos por dia durante uma semana.

Dois estudos não referiram o tempo de intervenção em dias^(18,19)^. O estudo de Rance et al.^(20)^ ocorreu nos cinco dias da semana, e no estudo de Rance et al.^(21)^ o período de intervenção foi de 8 a 10 dias. No estudo de Schafer et al.^(15)^ a intervenção ocorreu por 27 dias, incluindo os períodos com o SMR ligado (8 dias), SMR desligado (6 dias) e depois SMR novamente ligado (13 dias). No estudo de Schafer et al.^(16)^ foram entre 20 a 30 dias, período de dias similar ao estudo de Schafer et al.^(17)^, que foi em média de 30 dias. No estudo de Leung et al.^(22)^ o período foi de nove dias de treinamento auditivo em computador com duração de 20 a 30min, fazendo uso do SMR durante as sessões, e na escola por 15 dias (Figura 2).

No que se refere ao local da intervenção, sete estudos incluíram o ambiente escolar^(15-18,20-22)^, e destes, quatro realizaram a intervenção em casa concomitantemente^(16,17,21,22)^. O único estudo apenas em Laboratório, que não realizou a intervenção em ambiente escolar^(19)^, aplicou um ruído de fundo para simular o ruído escolar típico. Dois estudos filmaram todas as sessões de intervenção para análise dos comportamentos dos sujeitos^(18,19)^. No entanto, nos estudos incluídos não há detalhamento homogêneo das características físicas e acústicas dos ambientes (dimensões, revestimento, mobiliário), dois estudos referiram o controle do ruído de fundo^(18,21)^ durante a intervenção – inferior a 65 e 45 dB, respectivamente. O detalhamento da atividade pedagógica quando foi envolvido o ambiente escolar também foi heterogêneo, sendo referido em um estudo que os escolares estavam sentados com as cadeiras em círculo^(18)^ e em outro que havia uma atividade de grupo^(18)^.

### Objetivos e desfechos

Os objetivos e desfechos foram similares em todos os estudos, pois propuseram-se a estudar os eventuais efeitos do uso da tecnologia de SMR em crianças e adolescentes com TEA em aspectos auditivos, conforme apresentado na Tabela 2.

**Tabela 2:** Objetivos e desfechos dos estudos da revisão de escopo

Autor/ano/local	Objetivo	Desfecho
Keller et al.^(18)^ (2021), Estados Unidos da América	Verificar a eficácia do uso do SMR no desempenho de escuta funcional de pré-escolares com TEA e transtornos de linguagem	Observado melhor desempenho da escuta funcional das crianças e redução do tempo de resposta diante de comando verbal.
Keller^(19)^ (2021), Estados Unidos da América	Examinar os efeitos do uso do sistema SMR nas dificuldades de escuta em crianças com TEA e transtorno de linguagem grave	Melhora no desempenho auditivo de crianças com TEA minimamente verbais com SMR.
Rance et al.^(20)^ (2014), Austrália	Avaliar as habilidades de processamento mono e binaural em crianças com TEA	O Sistema FM demonstrou claros benefícios na audição e comunicação das crianças com TEA. Houve melhora na percepção de fala no ruído, na interação social e nos comportamentos em sala de aula.
Rance et al.^(21)^ (2017), Austrália	Avaliar o efeito da intervenção auditiva na resposta ao estresse em crianças escolares com TEA.	Melhora na percepção da fala em condições de escuta cotidiana (ruidosa), com melhor interação, e redução do estresse relacionado à audição.
Schafer et al.^(15)^ (2013), Estados Unidos da América	Verificar o benefício do Sistema FM em crianças com TEA e TDAH por meio de medidas de desempenho e reconhecimento de fala no ruído, e no comportamento observado em sala de aula;	Melhora no reconhecimento de fala no ruído e no comportamento durante as tarefas em sala de aula ao fazerem uso do SMR.
Schafer et al.^(16)^ (2014), Estados Unidos da América	Relatar o desempenho de reconhecimento de fala no ruído; compreensão auditiva; pelos participantes , pais e professores com MR	Melhora no reconhecimento de fala e na comunicação
Schafer et al.^(17)^ (2016), Estados Unidos da América	Avaliar os potenciais benefícios do MR em crianças com TEA.	Melhora na fala no ruído, atenção, memória auditiva, tolerância ao ruído e compreensão auditiva
Leung et al.^(22)^ (2021), Nova Zelândia	Explorar os efeitos do treinamento auditivo nas habilidades de percepção social de crianças com TEA	Melhora no desempenho comportamental nas medidas da percepção social, resultado eletrofisiológico mostrou alteração na atividade neural em resposta a mudanças pós intervenção

Fonte: Próprio autor

## DISCUSSÃO

Este estudo analisou e sintetizou oito artigos científicos, publicados em literatura internacional, com a finalidade de mapear os estudos com crianças e adolescentes com TEA que fizeram uso do SMR.

Os estudos encontrados se concentram em países desenvolvidos: EUA^(15-19)^, Austrália^(20,21)^ e Nova Zelândia^(22)^, com maior número nos EUA. Não foi encontrado nenhum estudo brasileiro. A não inclusão de pesquisas brasileiras nesta revisão pode ser um reflexo do uso e disponibilidade da tecnologia de MR no próprio Sistema Único de Saúde (SUS). Atualmente o acesso está regulamentado para a população com Deficiência Auditiva matriculada na rede de ensino^(23)^. Com os estudos escassos, não há ainda uma recomendação nacional, como há *guidelines* internacionais, para o uso do MR nessa população.

Pode-se considerar que as publicações incluídas são recentes, já que todos os estudos encontrados nesta revisão de escopo são da última década, sendo possível inferir que o uso do SMR em sujeitos com TEA ainda está sendo pouco explorado.

Os estudos investigaram crianças e adolescentes de diferentes faixas etárias. Um estudo^(18)^ interviu apenas em crianças pequenas entre três e quatro anos, enquanto os demais estudos^(15-21)^ incluíram crianças de seis anos até adolescentes. A faixa etária mais ampla dos estudos reflete nas diferenças metodológicas encontradas, uma vez que cada faixa etária tem suas especificações para seleção de tecnologias assistivas e do processo interventivo, de maneira mais global. A seleção de crianças maiores, e não pré-escolares como participantes da maior parte dos estudos dessa Revisão pode ser associada ao fato do uso do SMR pessoal ser desafiador em crianças pequenas - pode causar o efeito de oclusão, ou ainda acidentes com as olivas/receptores. Outro fator limitante seria a disponibilidade de uso do SMR em campo livre, por não ser indicado em ambientes reverberantes. Nesse aspecto de caracterização do espaço ambiental em que houve a intervenção do SMR, o detalhamento é heterogêneo entre os estudos. Apesar disso, constitui um relevante aspecto a ser considerado nos ambientes de intervenção e de ensino, uma vez que o controle do ruído e reverberação contribui positivamente na acessibilidade auditiva.

As alterações sensoriais apresentadas pelas crianças com TEA, como a hipersensibilidade aos sons ou a aceitação de usar dispositivos nas orelhas, dificuldades de linguagem e comunicação ou para relatar experiências pessoais por meio verbal ^(24,25)^, podem ter restringido o número de estudos com crianças menores de seis anos. Em pelo menos cinco estudos^(17,19-22)^ os adolescentes utilizaram SMR com receptor na orelha e não apresentaram resistência ao uso.

Dois estudos incluídos utilizaram o SMR em campo livre^(18,19)^. No estudo de Rance et al.^(21)^, seus participantes foram divididos em dois grupos de acordo com a idade. O grupo das crianças menores (6 anos) usaram SMR de campo livre, enquanto as crianças maiores (16 anos) fizeram uso de SMR com receptor na orelha, possivelmente pelos desafios referidos anteriormente. Estudos realizados por Schafer et al.^(3)^ e Feldman et al.^(4)^ usaram SMR pessoal em crianças a partir de seis anos até adultos jovens, assim, estes corroboram que o uso em crianças maiores seria facilitado no processo de intervenção, como também, mostram-se mais colaborativas com o uso do SMR pessoal em qualquer ambiente de intervenção.

A confirmação do diagnóstico de TEA é fundamental para a definição da conduta terapêutica, como também a indicação de tecnologia assistiva. Na presente revisão, o instrumento/avaliação diagnóstica para o TEA não foi reportado com clareza em um artigo^(15)^, mas os autores referem que a população do estudo foi oriunda de um serviço, sendo possível que o diagnóstico estivesse estabelecido previamente de acordo com os critérios adotados pela instituição. Ainda assim, a confirmação desse diagnóstico no momento da pesquisa poderia ser facilmente realizada com aplicação de protocolo de diagnóstico de TEA dos sujeitos de seu estudo. Conduta semelhante a esta foi realizada em outro estudo^(22)^, no qual realizaram a aplicação de protocolo CARS-2 para ratificar o diagnóstico de TEA dos participantes. Todos os outros estudos^(16-21)^ atestaram o diagnóstico de TEA através de instrumentos ou de acesso aos relatórios diagnósticos, mesmo quando houve relato dos pais e/ou escola sobre o diagnóstico. O nível de suporte dos sujeitos incluídos nos estudos não foi detalhado, o que não permite inferências acerca da indicação do uso do SMR na população com TEA a partir desse indicativo. A classificação do nível de suporte no TEA foi proposta no DSM-V em 2013, como os estudos incluídos eram de 2013 a 2021 é possível que a adoção destes critérios de classificação não tenha sido adotada nos estudos de anos iniciais, mas que poderiam ter agregado valor aos estudos mais recentes.

O desenvolvimento linguístico dos participantes é descrito como verbal. No entanto o nível de linguagem foi heterogêneo, havendo crianças com pouco repertório linguístico^(17-19)^, outras com maiores habilidades linguísticas^(17,20,21)^, e ainda aquelas que eram verbais, sem terem tido uma caracterização da linguagem^(15,16,22)^. A avaliação das habilidades verbais de crianças autistas é de grande relevância, pois elas apresentam, em sua maioria, dificuldades de linguagem e fala distintas em cada indivíduo^(26,27)^. Assim, a compreensão sobre se um indivíduo com TEA é verbal favorece a possibilidade de aplicação de questionários voltados ao participante da pesquisa referente ao uso do SMR, podendo contribuir na coleta de informações durante o uso do dispositivo, como foi realizado no estudo de Shafer et al^(17)^, no qual as crianças responderam ao questionário LIFE-R. A possibilidade de mensuração mais ampla dos resultados do uso da tecnologia assistiva pode fornecer um importante suporte não só no estabelecimento de critérios de indicação do SMR, como também no monitoramento terapêutico, favorecendo os ajustes da tecnologia durante o seu uso.

No que se refere a acuidade auditiva dos participantes, em um estudo a avaliação auditiva não foi realizada^(20)^, e em outro foi considerado apenas o relato de responsáveis^(19)^. Estas informações são relevantes e preocupantes, tendo em vista que mesmo perdas auditivas leves não identificadas podem gerar prejuízos tanto no desenvolvimento das habilidades auditivas, como também no processo de desenvolvimento da linguagem^(28)^. O diagnóstico da presença ou ausência de perda auditiva periférica é fundamental, uma vez que a indicação da tecnologia assistiva requer essa informação. Assim, na ausência de informações prévias da acuidade auditiva, deve ser priorizada a realização da triagem auditiva ou avaliação audiológica^(29)^. Todos os demais estudos^(15-17,21,22)^ confirmaram a audibilidade dos indivíduos, através de exames como emissões otoacústicas transientes (EOAT), emissões otoacústicas produto de distorção (EOAPD), pesquisa de limiares auditivos tonais, medidas em campo livre e/ou análise retrospectiva de prontuário. Além dessa mensuração, a verificação do próprio SMR através da medida de sua transparência, é essencial para favorecer a melhor e mais individualizada adaptação possível, embora sua aplicação não tenha sido referida nos estudos incluídos.

Durante o processo de intervenção, houve variabilidade no tempo de uso dia/sessão em minutos ou horas com SMR. Nos estudos de Schafer et al.^(16,17)^, Keller et al.^(18)^, Schafer et al.^(20,21)^ o tempo foi de 2 a 6 horas de intervenção por dia, enquanto que nos demais estudos esse tempo foi menor ou não referenciado^(15,19,22)^. É importante ressaltar que nenhum dos oito estudos referiram a confirmação do uso da tecnologia assistiva pelos professores/pais através de um acompanhamento descritivo diário de uso do SMR durante a intervenção. Esse registro seria facilitador ao acompanhamento do uso e efetividade da tecnologia, uma vez que depende do uso pelo falante principal – no caso dos estudos incluídos, professores e pais.

Entre os oito estudos, dois não referiram o tempo de intervenção^(18,19)^, e seis referiram de 8 a 10 dias (duas semanas) e máximo de 30 dias (6 semanas) de período de intervenção^(15-17,20-22)^.

Os benefícios e os critérios de indicação do SMR para a população com Deficiência Auditiva (DA) são conhecidos e bem descritos na literatura, cenário ainda não encontrado para a população com TEA. Nos estudos de Benítez-Barrera et al.^(5,6,30)^, foi realizada intervenção com SMR em crianças com DA durante o período de dois finais de semana, alternados com SMR e sem SMR. Tanto nos dois dias com SMR em casa por crianças com DA, como nos estudos com SMR e TEA aqui apresentados, de 8 até 30 dias de intervenção, foi possível observar benefício com a tecnologia assistiva, havendo melhora na percepção de fala em todos os estudos. Ainda assim, observando os impactos da melhora da acessibilidade auditiva e compreendendo as diferenças entre a DA e o TEA, podemos inferir que o maior tempo de intervenção beneficia o *input* auditivo através da tecnologia de SMR, pois a melhora na percepção de fala ao longo do tempo favorece o mecanismo de plasticidade auditiva^(31)^.

No que se refere ao ambiente, apenas um estudo realizou a intervenção exclusivamente em laboratório com ruído típico escolar^(19)^. A escola foi o principal ambiente escolhido pelos pesquisadores^(15,18,20)^ para o processo de intervenção, embora com limitações na descrição das atividades pedagógicas realizadas no período de observação – contexto que pode também interferir no ruído de fundo. Nesse sentido, dois estudos referem o controle do ruído de fundo^(18,21)^, no entanto o detalhamento das características do ambiente em termos físicos e acústicos é limitado e não há padrão de descrição.

A intervenção ocorreu em casa e/ou na escola em quatro estudos^(16,17,21,22)^. O uso do SMR em ambiente domiciliar ainda é pouco estudado, provavelmente pela ausência nas evidências que favorecem a viabilidade e a eficácia em ambiente domiciliar^(5)^, que conta com mais variáveis e os pesquisadores têm menor possibilidade de controle sobre elas. No entanto, por ser um ambiente em que as crianças ficam muito tempo durante a semana, a escola além de ser um ambiente de interação e onde há distratores ambientais^(32)^, torna-se o principal ambiente de intervenção e de observação do benefício do uso do SMR, com instrumentos que permitam acompanhar esse benefício.

Também é necessário que os professores estejam qualificados a novas estratégias que vão facilitar o processo de aprendizagem das crianças^(33)^. No estudo de Sposito et al.^(34)^, a falta de apoio dos professores foi citada como um desafio pelas crianças e adolescentes com DA para o uso do SMR em sala de aula.

Nas avaliações pré e pós uso do SMR nas crianças, foram aplicados diferentes instrumentos. Foi usado o teste ANL em um estudo^(17)^ para observar o nível de ruído que era aceitável com ou sem o uso do SMR. O estudo de Koiek et al.^(35)^, também aplicou o ANL em 93 crianças de ambos os sexos, entre 7-12 anos de idade, separadas em dois grupos, com e sem transtorno de aprendizagem e concluíram que o grupo com TEA toleram menos ruído de fundo. No estudo de Freyaldenhoven e Smiley^(36)^ foi avaliado o nível aceitável de ruído em 32 crianças entre 8-12 anos com sensibilidade auditiva normal. Foram avaliados o nível confortável de audição (MCLs) e os níveis máximos de ruído de fundo (BNLs) para conseguirem obter a ANL, e verificaram que o nível de ruído aceitável independe de sexo e da idade das crianças do estudo. Os resultados sugerem pesquisas com crianças com DA e ANL para o prognóstico do sucesso ou rejeição do aparelho de amplificação sonora individual (AASI). Estes estudos também referiram que o teste é aplicável em crianças de forma rápida^(35,36)^. Já o teste CNC foi usado apenas nos dois estudos de Rance et al.^(20,21)^. Trata-se de um teste antigo^(37)^ para avaliar a percepção de fala em sujeitos com ou sem dificuldades auditivas^(38-40)^.

A habilidade de processamento temporal auditivo foi avaliada com a técnica de Álcantara et al.^(41)^, que observou uma diferença das crianças com TEA de seu estudo quando comparadas com controle (pareados por idade e Quociente de Inteligência), sugerindo que as crianças com TEA apresentavam redução da sensibilidade de modulação temporal. Rance et al.^(20)^ lançou mão desta técnica, mostrando que houve menor sensibilidade das crianças menores com TEA às mudanças temporais. O teste LISN-S que avalia o processamento binaural foi aplicado unicamente por Rance et al.^(20)^, observando pobre integração das crianças com TEA. O teste (LISN-S) também foi relatado no estudo de Cameron et al.^(42)^ que avaliaram dez crianças com risco para Transtorno de Processamento Auditivo Central (TPAC), com resultados piores em todas as medidas de LISN-S para essas crianças do que para seus controles pareados por idade, concluindo que este teste seria promissor na bateria de testes de PAC.

Nesses três estudos, Schafer et al.^(15-17)^ usaram em comum o BKB-SIN, para avaliar o reconhecimento de fala em ruído^(43)^. O BKB-SIN foi aplicado no estudo de Ng et al.^(44)^ em grupos mistos composto por adultos sem DA, crianças sem DA e crianças com DA, demonstrando confiabilidade nos resultados. Outros estudos com crianças também fizeram uso deste teste em suas investigações^(45-49)^.

O teste LTC-2 avalia a compreensão auditiva e foi realizado no estudo de Schafer et al.^(16)^ sendo observado melhora nesta habilidade.

Os estudos de Keller et al.^(18)^ e Keller^(19)^ não utilizaram instrumentos padronizados para avaliar o reconhecimento da fala. Os autores adotaram um procedimento observacional da performance de escuta através de perguntas às crianças, com base em três categorias, e seguindo o nível de comunicação. Apesar de não terem usado um instrumento validado, conseguiram comprovar a melhora no reconhecimento da fala dessas crianças minimamente verbais. O uso de instrumentos validados é importante por apresentar padrões de normalidade que favoreceram a interpretação de resultados de forma clara. Embora os estudos incluídos tenham avaliado habilidades distintas, houve avanço na percepção de fala em todos os sujeitos com TEA que utilizaram o SMR.

Entre os questionários que foram aplicados, o CHAPS foi usado em três estudos desta revisão^(15-17)^. O LIFE também foi um instrumento escolhido e aplicado^(16,17,20)^ e o APHAB foi usado em dois estudos^(20,21)^ e, por fim, o CHILD foi aplicado em dois estudos^(16,17)^. Estes questionários têm como finalidade extrair informações sobre as habilidades e dificuldades de escuta no ambiente escolar e/ou em casa. Esses instrumentos foram também aplicados em estudos com sujeitos com TPA e uso de SMR, como triagem auditiva, em idosos com DA e uso de AASI, como também, em sujeitos com TEA^(50-55)^. A seleção do instrumento para colher informações sobre as habilidades e dificuldades auditivas é particularmente relevante na população com TEA não verbal, sendo a observação estruturada um importante balizador para esse público.

Os aspectos comportamentais foram avaliados com o CBCL e TRF no estudo de Rance et al.^(21)^ e uma caracterização comportamental no estudo de Schafer et al.^(15)^. O CBCL contribui na identificação de transtornos emocionais como depressão e ansiedade, na dificuldade de atenção, além dos comportamentos agressivos, havendo pesquisas com crianças sob custódia, problemas de ansiedade e afetivos e bipolaridade. TRF identifica comportamentos interferentes das crianças em ambiente escolar^(56-59)^. A melhora na acessibilidade auditiva pode igualmente modificar o tipo e frequência de comportamentos interferentes, sendo mais uma variável a ser observada.

O perfil sensorial foi apenas explorado em um estudo^(17)^, onde observaram que os participantes com TEA, quando usaram o SMR, apresentaram menor dificuldade na filtragem auditiva e na sensibilidade visual/auditiva. No estudo de Lyons-Warren et al.^(60)^, ao caracterizarem essas crianças com TEA com o uso do instrumento SSP, observaram entre os grupos que houve crianças que apresentaram alterações auditivas isoladas, e um outro grupo com diferenças na audição e paladar concomitantes. O estudo de O’Brien et al.^(61)^ corrobora estes achados em crianças com TEA, ao verificarem níveis elevados de hipersensibilidade auditiva/visual em comparação com os controles. Assim, o SSP é um instrumento amplamente utilizado nessa população^(62,63)^ e no estudo de Schafer et al.^(17)^ nesta revisão, ao comparar com e sem SMR, evidenciou melhora na condição auditiva com uso de SMR nesta população.

O nível de estresse foi estudado através da verificação de concentração do cortisol salivar nas crianças, que é considerado um importante biomarcador do estresse^(64,65)^. Estudos como de Tordjman et al.^(66)^ e Ogawa et al.^(67)^ têm sido realizados com crianças com TEA para avaliação dos níveis de cortisol, pois nestas crianças são observadas regulação diurna variável e maior reatividade, principalmente em crianças mais velhas^(68)^. Apenas um estudo desta revisão, o de Rance et al.^(21)^, estudou o estresse através desse biomarcador, sendo observado que o nível de cortisol estava elevado nas crianças que apresentavam pior percepção de fala gerando maior esforço auditivo, o que estaria provavelmente associado à maior nível de ansiedade para o desempenho das tarefas auditivas.

Foi usada a ACS em um dos estudos^(22)^, que é uma medida de percepção social^(69)^, sendo uma escala que pode ser realizada individualmente ou em grupo. Leung et al.^(22)^ verificaram que na comparação com uso do SMR e treino auditivo pelo computador por três semanas, houve melhora significativa na capacidade da percepção social, tanto que, crianças com TEA superaram o grupo controle (GC, que não recebeu intervenção) na categoria de nomeação de afeto e se equiparar às pontuações de prosódia de percepção social com os pares de crianças do GC. Estes resultados nos permitem inferir que o processo de intervenção foi benéfico para as crianças com TEA, e que favoreceu a melhora na percepção social.

No estudo de Leung et al.^(22)^ os autores realizaram a medida eletrofisiológica Mismatch *response* (*MMR*) nas crianças com TEA, e verificaram mudanças nas janelas de tempo pós intervenção, contudo os resultados mostraram que não ficou dentro do esperado como “normal” para o processamento auditivo. Assim, pode-se pensar que o tempo de intervenção foi insuficiente para normalizar esse processamento, contudo já trouxe benefícios às crianças do estudo quando comparado com o próprio desempenho na etapa pré intervenção, e ao serem comparadas com o GC pós intervenção. Ainda assim, estudos precisam ser realizados para corroborar esses achados com melhores condições de controle, pois o *MMR* é promissor na investigação da maturação auditiva de crianças, como é mencionado na revisão sistemática e de meta-análise realizada por Themas et al^(70)^. Além disso, o *MMR* pode estar relacionado bilateralmente no córtex auditivo e frontal, com lateralidade ao hemisfério esquerdo quando há processamento da fala^(71)^. Outras medidas eletrofisiológicas podem ainda ser aplicadas como marcadores de mudanças das habilidades auditivas, como as incluídas na bateria de avaliação do processamento auditivo^(72)^.

Os estudos^(15-22)^, apresentaram similaridades em seus objetivos, ou seja, o processo de intervenção com SMR na população com autismo foi a linha de base, e o desfecho da observação e caracterização dos achados pré e pós intervenção. A similaridade também aconteceu em relação aos resultados que verificaram a melhora na percepção da fala como principal benefício da intervenção com SMR. Contudo, os estudos aqui elencados indicaram outros possíveis benefícios que a tecnologia assistiva poderia favorecer, como nos aspectos comportamentais, sociais, redução do nível de estresse, modificação da resposta eletrofisiológica após uso do SMR e treinamento auditivo, e minimização do esforço auditivo.

## CONCLUSÃO

Os resultados da revisão de escopo demonstraram que crianças e adolescentes com TEA se beneficiaram com o processo de intervenção com a tecnologia assistiva de SMR melhorando a percepção da fala. Estes resultados contribuíram no processo de desenvolvimento destas crianças em ambientes escolares, familiares e sociais.

Ainda assim, mais pesquisas devem ser realizadas com um número amostral maior, com metodologias similares a fim de constituir evidências relativas aos critérios de indicação da tecnologia. O uso dessas ferramentas para mensurar de forma mais efetiva os impactos do *input* auditivo favorecido pelo uso do SMR pode evidenciar não apenas os resultados positivos referentes à percepção de fala como também impactos secundários, que já se mostram evidentes nos estudos desta revisão, mas que precisam de mais estudos que corroboram esses achados.

Assim, o SMR é promissor para uso nesta população, ao minimizar dificuldades na percepção de fala, propiciando benefícios secundários comportamentais, reduzindo esforço auditivo e estresse.

## Apêndice 1 Estratégia de pesquisa nas bases de dados

**Table:**

Database	Search (16th March 2023)
**Embase**	("Autism Spectrum Disorder" OR "Autism Spectrum Disorders" OR "Autistic Spectrum Disorder" OR "Autistic Spectrum Disorders" OR "Autistic Disorder" OR "Infantile Autism" OR "Autism" OR "Early Infantile Autism") AND ( "Sensory Aids" OR "Sensory Aid" OR "Self-Help Devices" OR "Self Help Devices" OR "Remote Microphone Systems" OR "Microphone System" OR "remote microphone technology" OR "Remote Microphone System" OR "FM system" OR "frequency modulation system")
**LILACS**	("Tecnologia Assistiva" OR "Dispositivos de Autoayuda" OR "Dispositivos Asistivos" OR "Dispositivos de Autoajuda" OR "Equipamentos Assistivos" OR "Equipamentos de Autoajuda" OR "Sistema de frequência modulada" OR "Sistema FM" OR "Microfone Remoto" OR "Assistive Technology" OR "Self-Help Devices" OR "Remote Microphone Systems" OR "Microphone System" OR "remote microphone technology" OR "Remote Microphone System" OR "FM system" OR "frequency modulation system" OR "remoto de micrófono" OR "sistema de frecuencia modulada" OR "tecnología de asistencia") AND (("Transtorno Autístico" OR "Autismo" OR "Autismo Infantil" OR "Síndrome de Kanner" OR "Transtorno do Espectro Autista" OR "Autism Spectrum Disorder" OR "Kanner's Syndrome" OR "Infantile Autism" OR "Autistic Disorder" OR "Transtorno del Espectro Autista"))
**PubMed**	("Autism Spectrum Disorder"[Mesh Terms] OR "Autism Spectrum Disorder" OR "Autism Spectrum Disorders" OR "Autistic Spectrum Disorder" OR "Autistic Spectrum Disorders" OR "Autistic Disorder" [Mesh Terms] OR "Autistic Disorder" OR "Kanner's Syndrome" OR "Kanner Syndrome" OR "Infantile Autism" OR "Autism" OR "Early Infantile Autism") AND ("Sensory Aids" [MeSH Terms] OR "Sensory Aids" OR "Sensory Aid" OR "Self-Help Devices" [MeSH Terms] OR "Self-Help Devices" OR "Self Help Devices" OR "Self-Help Device" OR "Remote Microphone Systems" OR "Microphone System" OR "remote microphone technology" OR "Remote Microphone System" OR "FM system" OR "frequency modulation system")
**Scopus**	("Autism Spectrum Disorder" OR "Autism Spectrum Disorders" OR "Autistic Spectrum Disorder" OR "Autistic Spectrum Disorders" OR "Autistic Disorder" OR "Autistic Disorder" OR "Kanner's Syndrome" OR "Kanner Syndrome" OR "Infantile Autism" OR "Autism" OR "Early Infantile Autism") AND ("Sensory Aids" OR "Sensory Aid" OR "Self-Help Devices" OR "Self Help Devices" OR "Self-Help Device" OR "Remote Microphone Systems" OR "Microphone System" OR "remote microphone technology" OR "Remote Microphone System" OR "FM system" OR "frequency modulation system")
**Web of Science**	("Autism Spectrum Disorder" OR "Autism Spectrum Disorders" OR "Autistic Spectrum Disorder" OR "Autistic Spectrum Disorders" OR "Autistic Disorder" OR "Kanner's Syndrome" OR "Kanner Syndrome" OR "Infantile Autism" OR "Autism" OR "Early Infantile Autism") AND ("Sensory Aids" OR "Sensory Aids" OR "Sensory Aid" OR "Self Help Devices" OR "Self-Help Device" OR "Remote Microphone Systems" OR "Microphone System" OR "remote microphone technology" OR "Remote Microphone System" OR "FM system" OR "frequency modulation system")
**Google Scholar**	("Autism" OR"Autism Spectrum Disorder" OR "Autistic Disorder") AND (“remote microphone system” OR “frequency modulation system” OR "sensory aids")
**ProQuest**	("Autism Spectrum Disorder" OR "Autism Spectrum Disorder" OR "Autism Spectrum Disorders" OR "Autistic Spectrum Disorder" OR "Autistic Spectrum Disorders" OR "Autistic Disorder" OR "Autistic Disorder" OR "Kanner's Syndrome" OR "Kanner Syndrome" OR "Infantile Autism" OR "Autism" OR "Early Infantile Autism") AND ("Sensory Aids" OR "Sensory Aids" OR "Sensory Aid" OR "Self-Help Devices" OR "Self-Help Devices" OR "Self Help Devices" OR "Self-Help Device" OR "Remote Microphone Systems" OR "Microphone System" OR "remote microphone technology" OR "Remote Microphone System" OR "FM system" OR "frequency modulation system")

## Apêndice 2 Razão do critério de exclusão

**Table:**

Autor, Ano	Razão da exclusão
Dunn A, James P, Pelosi A, Sorensen E, Oleson J; 2021	**1**
Feldman JI, Thompson E, Davis H, Keceli-Kaysili B, Dunham K, Woynaroski T. et al.; 2022	**2**
Hess KL, Morrier MJ, Heflin, Heflin LJ, Ivey ML; 2008	**3**
Rance G; 2013	**3**
Schafer, E C; Gopal, K V; Mathews, L; Thompson, S; Kaiser, K; McCullough, S; Jones, J; Castillo, P; Canale, E; Hutcheson, A; 2019	**2**
Teleaudiology Today, 2022	**3**
Westby, C, 2014	**3**
1) Estudos que não sejam de intervenção; 2) Estudos com adultos/idoso; 3) revisões, artigos de opinião, posters, letters e resumos de conferências.	
Referências:	
1. Dunn A, James P, Pelosi A, Sorensen E, Oleson J. Managing listening difficulties in patients with ASD and normal hearing sensitivity. Hearing Review. 2021.	
2. Feldman JI, Thompson E, Davis H, Keceli-Kaysili B, Dunham K, Woynaroski T et al. Remote microphone systems can improve listening-in-noise accuracy and listening effort for youth with autism. Ear Hear. 2022;43(2):436-447.	
3. Hess KL, Morrier MJ, Heflin, Heflin LJ, Ivey ML. Autism treatment survey: services received by children with autism spectrum disorders in public school classrooms. J Autism Dev Disord. 2008;38:961-971.	
4. Rance G. In autism study, FM system enhances listening, attention, and behavior. Journal club. 2013;66(7)4-5.	
5. Schafer EC, Gopa KV, Mathews L, Thompson S, Kaiser K, McCullough S et al. Effects of auditory training and remote microphone technology on the behavioral performance of children and young adults who have autism spectrum disorder. J Am Acad Audiol. 2019;30:431-443.	
6. Teleaudiology today [internet]. Can Remote Microphone Systems Improve Listening-in-noise Accuracy in Children with Autism? The Hearing Journal. 2022*;*75(3):10. [cited 2023 nov 15]. available from: https://journals.lww.com/thehearingjournal/Fulltext/2022/03000/Can_Remote_Microphone_Systems_Improve.7.aspx	
7. Westby, C. FM; 2014. Systems with students with autism spectrum disorders or attention deficit hyperactivity disorder. [cited 2023 nov 15] [about 1 screens] Available from: https://journals.sagepub.com/doi/10.1177/1048395014527568b.
